# Altered Executive Function in Suicide Attempts

**DOI:** 10.1192/j.eurpsy.2022.343

**Published:** 2022-09-01

**Authors:** J. Fernández, S. Alberich, I. Zorrilla, I. González-Ortega, M.P. López, V. Pérez-Solà, E. Vieta, A.M. González-Pinto, P.A. Saiz

**Affiliations:** 1 Asociación Instituto de Investigación Sanitaria BIOARABA. Universidad del País Vasco, Departamento de Neurociencias, Psychiatry, Vitoria-Gasteiz , Spain; 2 Asociación Instituto de Investigación Sanitaria BIOARABA, Psychiatry, Vitoria-Gasteiz , Spain; 3 Parc de Salut Mar, Institut De Neuropsiquiatria I Addiccions, Barcelona, Spain; 4 Hospital Clínic de Barcelona, Bipolar And Depressive Disorders Unit, Institute Of Neuroscience, Barcelona, Spain; 5 University of Oviedo, Department Of Psychiatry, oviedo, Spain

**Keywords:** Executive function, cognition, major depressive disorder, Suicide

## Abstract

**Introduction:**

Executive function organizes and directs behaviour but alterations in this cognitive domain can lead to inaccurate perception, interpretation and response to environmental information, which could be a risk factor for suicide.

**Objectives:**

To explore executive function performance of depressed recent suicide attempters in comparison to depressed past suicide attempters, depressed non-attempters and healthy controls.

**Methods:**

96 participants from the Psychiatry Department of the Araba University Hospital-Santiago were recruited as follows: 20 patients with a recent suicide attempt (<30days) diagnosed with a Major Depressive Disorder (MDD), 33 MDD patients with history of attempted suicide, 23 non-attempter MDD patients and 20 healthy controls. All participants underwent a clinical interview and neuropsychological assessment on executive function with the Wisconsin Sorting Card Test. Backward multiple regressions were performed adjusting for significant confounding variables. For group comparisons ANOVA test and Bonferroni post hoc test were performed with p<0.05 significance level.

**Results:**

Patient groups did not differ regarding severity of depression. All patient groups performed significantly worse than healthy controls on executive function. Adjusted comparisons between patient groups indicated that recent suicide attempters had a poorer performance in this cognitive domain in comparison to both depressed lifetime attempters and depressed non-attempters (B=0.296, p=0.019 and B=0.301, p=0.028 respectively).

**Conclusions:**

Executive function performance is altered in recent suicide attempts. As impaired executive function can be a risk factor for suicide, preventive interventions on suicide should focus on its assessment and rehabilitation.

**Disclosure:**

No significant relationships.

